# Assessment of Knowledge, Attitude, and Preventive Behavior Regarding Toxoplasmosis among Females in Riyadh, Saudi Arabia: A Cross-Sectional Study

**DOI:** 10.3390/ijerph21081065

**Published:** 2024-08-14

**Authors:** Jehad A. Aldali, Ala M. Aljehani, Emadeldin M. Elsokkary, Fouz L. Alkhamis, Norah M. Bin Khathlan, Hind H. Alhadban, Hala K. Alkhathlan

**Affiliations:** 1 Department of Pathology, College of Medicine, Imam Mohammad Ibn Saud Islamic University (IMSIU), Riyadh 13317, Saudi Arabia; amaljehani@imamu.edu.sa; 2 Department of Psychology, College of Social Sciences, Imam Mohammad Ibn Saud Islamic University (IMSIU), Riyadh 13317, Saudi Arabia; emelsokkary@imamu.edu.sa; 3 College of Medicine, Imam Mohammad Ibn Saud Islamic University (IMSIU), Riyadh 13317, Saudi Arabia443012768@sm.imamu.edu.sa (H.H.A.);

**Keywords:** toxoplasmosis, knowledge, attitude, preventive behavior, females in Saudi Arabia

## Abstract

Toxoplasmosis, a prevalent parasitic zoonotic disease, is influenced by various factors such as the climate, dietary habits, and hygiene practices. This study aimed to evaluate the knowledge, attitudes, and preventive behaviors regarding toxoplasmosis among females in Riyadh, Saudi Arabia. Utilizing a bilingual Google form, a cross-sectional online survey was distributed in both Arabic and English, and it was conducted between 11 January 2024 and 4 March 2024. Statistical analysis was conducted using SPSS Version 27, with a *p*-value ≤ 0.05 indicating significant qualitative data. The data were analyzed using descriptive statistics and the chi-square test. A total of 533 participants were included in the study. Participants aged 18–25 years old constituted the largest group (70.4%), with those aged 26–40 years old accounting for 14.4% and ages 41–60 years old comprising 15.2%. Among the participants, 76.4% were unmarried, and 21.4% were pregnant. Notably, 79.2% of participants reported being unaware of toxoplasmosis, with only 9.0% gaining awareness from doctors and a mere 3.6% from awareness campaigns. Any understanding of the disease’s severity and causative factors was limited to 15.9%. Despite a generally positive attitude towards preventive measures, significant correlations were found between toxoplasmosis and age (*p*-value 0.093), as well as the consumption of medium-cooked meat (*p*-value 0.008). Other variables, such as social status, cat ownership, handwashing before meals, and washing fruits and vegetables did not show significant correlations. Diet and hygiene practices notably impact toxoplasmosis transmission. In Riyadh, 79% of participants did not own cats, and 67.7% avoided undercooked meat. However, 6.7% used unfiltered water, and 8.4% did not wash their hands after handling raw meat and vegetables. The study concludes that there is insufficient knowledge regarding toxoplasmosis among females in Riyadh. Despite low knowledge, there is a neutral to slightly positive attitude and a willingness to learn and adopt preventive measures when informed. With better education, attitudes towards toxoplasmosis could improve due to a desire to learn and act. While general hygiene practices were favorable, specific preventive behaviors for toxoplasmosis need enhancement to reduce infection risks.

## 1. Introduction

Toxoplasmosis is induced by the parasite *Toxoplasma gondii* (*T. gondii*), affecting various warm-blooded animals, including humans, with cats serving as the primary host for sexual reproduction [1]. Studies show that approximately one-third of the global population is impacted by *T. gondii* [2]. Human infection commonly occurs through the ingestion of oocysts present in food or water contaminated with cat feces, or by consuming tissue cysts in undercooked meat. Other modes of transmission include congenital passage, organ transplantation, and blood transfusion [3].

Initial infection with *T. gondii* in healthy individuals, including pregnant women, typically remains asymptomatic. Manifested symptoms, when present, are general and may include malaise, fever, headache, lymphocytosis, sore throat, and muscle pain. Swollen lymph nodes, primarily in the neck, represent a common sign. In some cases, patients may experience an illness resembling mononucleosis, characterized by a rash, along with an enlarged liver and spleen [4,5].

Conversely, toxoplasmosis in immunocompromised individuals typically manifests as toxoplasmic encephalitis [6], occasionally accompanied by pneumonia. Additionally, isolated ocular toxoplasmosis may result from the reactivation of untreated congenital infections or occur in a limited number of individuals with acquired infections. This condition can lead to various ocular complications, such as choroidal neovascularization, cataracts, glaucoma, optic nerve atrophy, and retinal detachment, particularly in children [7].

There have been rare instances where females become infected with *T. gondii* before conception [8]. Multiple sources can transmit the parasites to females, including contaminated food, water, or cat feces. Females infected with *T. gondii* before conception may pass the infection to the fetus during pregnancy [8].

*T. gondii* has evolved mechanisms to evade immune responses, including the formation of tissue cysts, immunomodulation, and chronic infection, facilitating the establishment of latent infection [9]. Pregnancy-induced immune suppression, attributed to factors like hormonal changes, placental influences, microbial colonization, and regulatory T cell expansion, creates an environment for the rapid reactivation and proliferation of *T. gondii* [10,11]. Monitoring IgG antibodies through serological tests is vital to detect and prevent congenital toxoplasmosis [8].

During the first trimester of pregnancy, crucial fetal organogenesis occurs, affecting the development of major organs and structures. Disruptions during this phase may lead to severe consequences, such as intrauterine growth restriction, preterm birth, or spontaneous abortion, particularly if exposure occurs early in pregnancy [12].

The prevalence of toxoplasmosis in Asian countries varies, with rates ranging between 23% and 45% in pregnant women in Malaysia, 1.4% and 21.7% in Thailand, and up to 17.2% in Singapore. Notably, the Arabian Peninsula exhibits differences in seropositivity rates, with the UAE displaying the lowest rate at 22.9% and Kuwait the highest at 45.7% [13]. In Saudi Arabia, studies indicate prevalence rates ranging from 29.5% to 35.6% among pregnant women [14,15], with varying rates reported in different regions such as Jeddah (61.4%), Al Ahsa (51.4%), Aseer (38.8%), Riyadh (38%) [16], and Makkah (35.6%) [17].

Furthermore, a study highlighted that toxoplasmosis was the second leading cause of death from food-borne diseases in the USA, accounting for an estimated 327 deaths. It was also the fourth leading cause of hospitalizations due to food-related illnesses, with 4428 hospitalizations reported in the mid-to-late 2000s [14].

Toxoplasmosis is a major public health issue facing pregnant women and immunocompromised individuals, but data on women’s awareness, attitudes, and preventive practices in Riyadh, Saudi Arabia, are lacking. Toxoplasmosis is known to be harmful to fetal health, but there is little local research on women’s knowledge, attitudes, and behaviors. Cultural, educational, and socioeconomic factors that may influence knowledge and behavior in Riyadh are poorly understood due to the literature’s focus on general awareness in larger geographic areas or specific subpopulations. This gap hinders the development of public health interventions and population-specific educational campaigns. Therefore, this study seeks to fill this knowledge gap by assessing women’s awareness, attitudes, and behaviors in Riyadh regarding toxoplasmosis to improve public health and education.

This study aims to investigate the evaluation of the knowledge, attitudes, and preventive behaviors of females in Riyadh, Saudi Arabia.

## 2. Methods and Materials

### 2.1. Study Design and Settings

This cross-sectional study was conducted at the Department of Pathology, College of Medicine, Imam Muhammad bin Saud Islamic University, located in Riyadh, Saudi Arabia. The research took place from 11 January 2024 to 4 March 2024 to ensure sufficient data collection.

### 2.2. Study Participants

The study participants were members of the general population residing in the Riyadh province of Saudi Arabia. The participants included a diverse group of female students, teachers, and technicians, providing a broad spectrum of data. A portion of the participants were cat owners, while others were not, allowing for comparative analysis between these groups. The research team conducted visits to a range of locations, such as schools, universities, and student councils, in the Riyadh region of Saudi Arabia, to gather the contact information of the participants.

### 2.3. Sample Size Calculation

The study sample size was calculated using power calculator “Raosoft” [18]. The regional authorities of the Riyadh province of Saudi Arabia have a population of approximately eight million individuals, with a 49% female population. A sample size of 385 participants was deemed adequate to achieve a 95% confidence level with a 5% margin of error. Nevertheless, 582 individuals participated in the current investigation.

### 2.4. Inclusion and Exclusion Criteria

These criteria ensure the study population is relevant and appropriate for answering the research questions while minimizing confounding factors that could affect the findings.

Inclusion criteria:

- Gender: female participants only.
- Location: residents of Riyadh, Saudi Arabia.
- Consent: willingness to participate in the study.
- Language: ability to understand and respond in the language(s) in which the survey or assessment is conducted (i.e., Arabic, English).

Exclusion criteria:

- Non-residents: individuals not residing in Riyadh.
- Males: excluding male participants, as the study is specific to females.
- Language barriers: inability to understand survey or assessment language, which could lead to misinterpretation and inaccurate responses.
- Incomplete surveys: participants who do not complete the survey or assessment in full.

### 2.5. Study Questionnaire Development

A cross-sectional study employed an online survey and disseminated it through a bilingual (Arabic and English) Google form. The survey targeted female residents of Riyadh, Saudi Arabia. The link to the Google form used for this survey was as follows: “https://docs.google.com/forms/d/e/1FAIpQLSdjC__7YR1-NlDLkA5hhTysJAPimjBS-II9q9DmQZtl1uGuvA/viewform (11 January 2024)”. All authors reviewed and offered feedback on survey sections, suggesting edits as necessary.

The online survey was created and distributed on various social media platforms, including WhatsApp and Twitter, complemented by targeted emails directed towards public health and university staff. The online questionnaire commenced with a requirement for participants to read a comprehensive description that included the study intention and voluntary participation. It also ensured anonymity before assenting to an electronic consent form. For any participants’ concerns, the study researchers directed users to an email account. After the recipients conducted the questionnaire, the data were compiled anonymously and securely stored in a designated file. The closed-structured online questionnaire had four sections. While the first division initiated with a consent form for agreement, the second section investigated respondents’ demographical data, such as gender, age, nationality, educational rank, social status, pregnancy state, and toxoplasmosis infection situation. The third section explored the population’s knowledge of toxoplasmosis, like toxoplasmosis background, toxoplasmosis severity, information resources, and risk factors. The last division examined the understanding of the symptoms and the transmission of toxoplasmosis. The sample size calculation for that questionnaire using “raosoft.com (10 January 2024)” indicated three hundred and eighty-five participants as an adequate sample size to achieve a ninety-five percent confidence level, a five percent margin of error, and a fifty percent population proportion.

### 2.6. Statistical Analysis

The statistical analysis was conducted using SPSS Version 27. The data analysis involved both descriptive and inferential statistics. Descriptive statistics were used to summarize the demographic characteristics and general responses of the participants. These included frequencies and percentages.

For inferential statistics, Pearson chi-square tests were employed to identify significant relationships and associations between categorical variables. The chi-square test was used to examine the associations between awareness of toxoplasmosis and various demographic and behavioral factors. Specifically, a *p*-value ≤ 0.05 was considered to indicate statistical significance.

### 2.7. Ethical Approval

The research was reconsidered and authorized by Imam Muhammad bin Saud Islamic University’s Institutional Review Board (IRB) committee with project digit 576-2023, validated on 9 January 2024. The IRB-confirmed study was entitled, “The Prevalence of Awareness of Toxoplasmosis among Females in the Riyadh Region, Kingdom of Saudi Arabia: A Cross-Sectional Study”.

## 3. Results

This study involved a total of 533 participants. The bulk of participants, including 375 (70.4%), were within the age range of 18–25 years old. Participants aged 26–40 years old accounted for 77 (14.4%), while those aged 41–60 years old made up 81 (15.2%). Regarding social status, the majority of participants were unmarried, representing 407 (76.4%), while 125 (23.5%) were married. In terms of pregnancy status, 114 (21.4%) reported being pregnant, and 11 (2.1%) were not pregnant (Table 1).

Table 2 presents the participants’ background information on toxoplasmosis. Among the respondents, 422 (79.2%) indicated that they did not know about toxoplasmosis, while 111 (20.8%) reported being aware of it. Regarding the severity of the disease or the factors leading to toxoplasmosis, 436 (81.8%) had never known about toxoplasmosis. There was no response for 12 (2.3%) on this question. Meanwhile, whereas 85 (15.9%) acknowledged that they were aware of these aspects, in terms of information sources, the majority, 422 (79.2%), stated that they had never known about toxoplasmosis from any resource. Among those who had some information, 48 (9.0%) obtained it from doctors, 44 (8.3%) from social media, and 19 (3.6%) from awareness campaigns.

We are interested in the knowledge of the surveyed population regarding toxoplasmosis. Participants who were infected with toxoplasmosis are presented in Table 3. The vast majority of participants, 527 (98.9%), had not contracted toxoplasmosis, with only 6 (1.1%) having been infected. Among those infected, 2 (0.4%) reported recent, acute, and chronic infections, and all participants who were infected with toxoplasmosis had consumed medications for the infection.

Potential participants should have a history of toxoplasmosis or be newly infected. According to Table 4, there are 451 (84.6%) participants who do not own a cat as a pet, whereas 82 participants (15.4%) do. Regarding the cleaning of cat boxes, 35 (6.6%) of cat owners clean the boxes daily, 31 (5.8%) clean them every two days, and 18 (3.4%) clean them more frequently. A significant portion, 441 (82.7%), do not raise cats. For the annual examination of cats, 76 (14.3%) perform this test annually, 16 (3.0%) do not, and 439 (82.4%) do not raise cats in the first place. When asked about washing hands before eating, 317 (59.5%) use a combination of water and soap, 171 (32.1%) use water alone, and 45 (8.4%) do not wash their hands at all. Regarding washing fruits and vegetables before eating, 426 (79.9%) wash them with water only, 98 (18.4%) use water and soap, and 9 (1.7%) do not wash them at all. When it comes to eating medium-cooked meat, 386 (72.4%) never eat it, 92 (17.3%) rarely eat it, 48 (9.0%) sometimes eat it, and 7 (1.3%) often eat it. The data suggest a strong adherence to proper hygiene protocols among the participants, with the majority washing hands and produce regularly, and a significant portion avoiding medium-cooked meat.

Table 5 shows the analysis and explores the relationships between having toxoplasmosis and several variables using the chi-square test. The variables include age, social status, raising cats, washing hands before eating, washing fruits and vegetables before eating, and eating medium-cooked meat.

For the variable age, among participants aged 18–25, 2 individuals (33.3%) had toxoplasmosis, while 373 individuals (70.8%) did not, making up 70.4% of the total participants. In the age group 26–40, 3 individuals (50.0%) had toxoplasmosis compared to 74 individuals (14.0%) who did not, making up 14.4% of the total. In the age group 41–60, 1 individual (16.7%) had toxoplasmosis, while 80 individuals (15.2%) did not, making up 15.2% of the total. The chi-square value was 6.501, with a *p*-value of 0.039, indicating a significant relationship between age and having toxoplasmosis.

These analyses highlight a significant correlation between age and having toxoplasmosis, whereas no significant correlation was found between social status and having toxoplasmosis.

For raising cats, 1 participant (16.7%) who had toxoplasmosis raised cats, compared to 81 participants (15.4%) who did not have toxoplasmosis, and 5 participants (83.3%) who had toxoplasmosis did not raise cats, compared to 446 participants (84.6%) who did not have toxoplasmosis. The chi-square value was 0.008, and the *p*-value was 0.930, indicating that this relationship is not significant.

When asked about washing hands before eating, 0 participants (0.0%) who had toxoplasmosis did not wash their hands, compared to 45 participants (8.5%) who did not have toxoplasmosis; 2 participants (33.3%) who had toxoplasmosis washed their hands with water only, compared to 169 participants (32.1%) who did not have toxoplasmosis; and 4 participants (66.7%) who had toxoplasmosis washed their hands with water and soap, compared to 313 participants (59.4%) who did not have toxoplasmosis. The chi-square value was 0.568, and the *p*-value was 0.753, indicating that this relationship is not significant.

Regarding washing fruits and vegetables before eating, 0 participants (0.0%) who had toxoplasmosis did not wash their fruits and vegetables, compared to 9 participants (1.7%) who did not have toxoplasmosis; 3 participants (50.0%) who had toxoplasmosis washed their fruits and vegetables with water only, compared to 423 participants (80.3%) who did not have toxoplasmosis; and 3 participants (50.0%) who had toxoplasmosis washed their fruits and vegetables with water and soap, compared to 95 participants (18.0%) who did not have toxoplasmosis. The chi-square value was 4.081, and the *p*-value was 0.130, indicating that this relationship is not significant.

Concerning eating medium-cooked meat, 3 participants (50.0%) who had toxoplasmosis never ate medium-cooked meat, compared to 383 participants (72.7%) who did not have toxoplasmosis; 1 participant (16.7%) who had toxoplasmosis rarely ate medium-cooked meat, compared to 91 participants (17.3%) who did not have toxoplasmosis; 1 participant (16.7%) who had toxoplasmosis sometimes ate medium-cooked meat, compared to 47 participants (8.9%) who did not have toxoplasmosis; and 1 participant (16.7%) who had toxoplasmosis often ate medium-cooked meat, compared to 6 participants (1.1%) who did not have toxoplasmosis. The chi-square value was 11.710, and the *p*-value was 0.008, indicating a significant relationship.

In summary, significant correlations were found between having toxoplasmosis and age, as well as the consumption of medium-cooked meat. Other variables, such as social status, raising cats, washing hands before eating, and washing fruits and vegetables, did not show significant correlations.

## 4. Discussion

The prevalence of toxoplasmosis is widely recognized as one of the most widespread parasitic zoonotic diseases globally [19]. Various factors contribute to the spread of toxoplasmosis, including climatic conditions and dietary, economic, social, or cultural habits. Additionally, practices such as consuming undercooked meats, contaminated water or food, unboiled camel and cow milk, and poor hand hygiene increase the incidence of toxoplasmosis [20,21,22]. Countries with arid and colder weather, such as Riyadh, tend to have lower infection rates. Furthermore, this study reveals low rates of awareness and infection status among females in Riyadh, Saudi Arabia.

There were five hundred and thirty-three participants in the study; most of them were young adults between the ages of eighteen and twenty-three. Most of the participants were single. Among the respondents, the knowledge level about toxoplasmosis was clearly low; only a few received awareness from doctors and had very meagre knowledge from awareness campaigns. Moreover, knowing the severity and causal elements of the illness was even more restricted; just a very small fraction of people were informed. Though most people have a good attitude towards preventative actions, toxoplasmosis and age as well as medium-cooked meat consumption showed strong correlations. Not showing any notable correlations were other factors including social level, cat ownership, hand washing before meals, and washing fruits and vegetables. The transmission of toxoplasmosis is clearly influenced by diet and hygienic standards. Many of the participants in Riyadh skipped undercooked meat and did not own cats. Few of them, however, drank pure water, and few did not wash their hands after handling vegetables and raw meat.

These findings are consistent with previous cross-sectional community-based studies in Saudi Arabia [23] and globally [24,25], suggesting a need for increased awareness campaigns. Unlike the study of Jazan, which indicates that awareness of toxoplasmosis increases with age [16], this study found no such correlation. The study demonstrates a higher prevalence of the awareness of toxoplasmosis severity and leading factors compared to the Al-Ahssa study, which shows lower rates of awareness. In comparison to studies in Al-Ahssa and Morocco, where education was the primary information source [19,26], female participants showed positive attitudes toward toxoplasmosis. Despite low awareness, disease-savvy participants sought information from doctors and social media. This indicates a neutral to slightly positive attitude and a willingness to learn and take preventive measures when informed. With better education, attitudes toward toxoplasmosis may improve due to a desire to learn and act.

Only a small number of participants reported having any form of the infection, whether it was acute, chronic, or recent. This indicates that almost all of the participants had not been infected with toxoplasmosis to begin with. Medication was consistently used as a treatment method for those who were infected with the parasite. In the present study, the vast majority of females in Riyadh tested negative for toxoplasmosis, while only a minority tested positive. These findings suggest that toxoplasmosis is uncommon within the examined population compared to a study conducted on female university students in Jazan, Saudi Arabia [16]. Additionally, the number of infected females in Riyadh is far less than the reported cases among female students in Al-Ahssa, Saudi Arabia [17].

The female participants’ preventive behaviors were inconsistent, but they tended to be more optimistic regarding their general hygiene practices. The majority of individuals consistently cleaned fruits and vegetables with water and washed their hands before eating. Nevertheless, there is still room for improvement in the dietary habits of some participants, as they occasionally consume medium-cooked meat, which is a risk factor for toxoplasmosis. Additionally, the practices of cat ownership were suboptimal, as only a small percentage of cat owners conducted annual examinations and conducted regular cleanings. Although the general hygiene behaviors were commendable, the risk of infection must be effectively reduced through the implementation of specific preventive practices specifically related to toxoplasmosis.

Another significant finding regarding knowledge of toxoplasmosis symptoms and transmission is that most participants were unsure if it is always symptomatic and whether it could be transmitted from a pregnant woman to her fetus, whereas the study of Jazan shows higher rates of knowledge about these aspects [16]. The results emphasize the importance of raising awareness, as it leads to early detection and reduces the risk of a mother-to-child transmission of the infection [26].

Additionally, the study revealed that 8.6% of respondents had cats in their houses and did not regularly clean their litter boxes, putting them at risk of toxoplasmosis. Furthermore, 2.9% of cat owners do not seek medical examinations for their pets. Infected cats can generate massive amounts of cysts, an estimated 100 million eggs per cat, making them a primary source of environmental contamination and allowing the parasite to continue its life cycle.

Analyzing the relationships between toxoplasmosis and various variables using the chi-square test revealed some notable findings. Age showed a significant correlation with toxoplasmosis infection, indicating that the likelihood of having toxoplasmosis varied with age. However, social status did not show a significant relationship with the infection. Raising cats also did not significantly correlate with toxoplasmosis infection, suggesting that cat ownership alone was not a determining factor. Similarly, washing hands before eating and washing fruits and vegetables before eating was not significantly associated with having toxoplasmosis, indicating that these hygiene practices did not substantially impact the infection rates among the participants. Interestingly, a significant relationship was found between the consumption of medium-cooked meat and toxoplasmosis infection. This finding suggests that dietary habits, particularly the consumption of undercooked meat, could play a critical role in the likelihood of contracting toxoplasmosis.

### 4.1. Study Limitations

The study evaluating the knowledge, attitude, and preventive behavior toward toxoplasmosis among females in Riyadh, Saudi Arabia, has multiple constraints. The cross-sectional design of the study limits the ability to draw causal inferences, and the use of self-reported data may introduce response bias. The research is limited by a small sample size and being conducted exclusively in Riyadh, which might not accurately reflect the entire Saudi Arabian population. Future research should focus on examining the level of knowledge about toxoplasmosis among women in different areas of Saudi Arabia and finding the best strategies to increase awareness and control the transmission of the disease. Another limitation is that the study only looked at women in Riyadh, so it cannot be used for other regions or demographics. Due to non-response bias, results could be skewed, and knowledge assessment tools might not adequately reflect the intricacy of the concepts. Finally, a thorough comprehension of the topic may be obstructed by a lack of longitudinal data and unconsidered environmental factors.

### 4.2. Conclusions

The study concludes that there is insufficient knowledge about toxoplasmosis among females in Riyadh. Despite this low awareness, participants demonstrated a neutral to slightly positive attitude and a willingness to learn and adopt preventive measures when informed. Enhanced education could significantly improve attitudes toward toxoplasmosis, leveraging the participants’ desire to learn and act. Although general hygiene practices were favorable, specific preventive behaviors related to toxoplasmosis require improvement to reduce infection risks. The findings highlight the need for targeted awareness campaigns to educate females on the seriousness, risk factors, transmission, and symptoms of toxoplasmosis. Notably, significant correlations were found between toxoplasmosis and factors such as age and the consumption of medium-cooked meat, while other variables like social status, cat ownership, and handwashing before eating and washing fruits and vegetables did not show significant correlations.

## 5. Future Research

Future research should prioritize longitudinal studies in Riyadh to assess the long-term impact of educational interventions on knowledge and preventive behaviors related to toxoplasmosis among women. In addition, there is a need to explore how sociocultural factors and healthcare-provider interactions influence these attitudes and behaviors, with a particular focus on high-risk groups such as pregnant women. At a global level, cross-cultural comparative studies can shed light on how different cultural contexts shape knowledge and practices around toxoplasmosis, helping to identify global versus region-specific challenges. Large-scale epidemiological surveys are also essential to map awareness and incidence rates, thereby informing global public health strategies and resource allocation.

## Figures and Tables

**Table 1:** The demographic characteristics of the study participants (*n* = 533).

Variables		Frequency	Percentages %
**Age**	18–25	375	70.4%
	26–40	77	14.4%
	41–60	81	15.2%
**Social status**	NA	1	0.2%
	Unmarried	407	76.4%
	Married	125	23.5%
**Got pregnant**	NA	1	0.2%
	I am not married	407	76.4%
	No	11	2.1%
	Yes	114	21.4%

NA—not available.

**Table 2:** Background information about toxoplasmosis.

Variables		Frequency	Percentages %
**Know about toxoplasmosis**	No	422	79.2%
	Yes	111	20.8%
**Know about the severity of that disease or the factors leading to toxoplasmosis**	NA	12	2.3%
	No	436	81.8%
	Yes	85	15.9%
**Information resources**	I have never known about toxoplasmosis	422	79.2%
	Awareness campaigns	19	3.6%
	Social media	44	8.3%
	The doctor	48	9.0%

NA—not available.

**Table 3:** Participants who are infected with toxoplasmosis.

Variables		Frequency	Percentages %
**Got toxoplasmosis?**	No	527	98.9%
	Yes	6	1.1%
**Infection state**	I have not got toxoplasmosis	527	98.9%
	Infected recently	2	0.4%
	Acute infection	2	0.4%
	Chronic infection	2	0.4%
**Consume any medications for toxoplasmosis infection?**	I have not got toxoplasmosis	527	98.9%
	Yes	6	1.1%

**Table 4:** Individuals caring for cats adhere to proper hygiene protocols to mitigate disease transmission.

Variables		Frequency	Percentages %
**Keep cats as pets**	Yes	82	15.4%
	No	451	84.6%
**Clean cat box**	Every day	34	41.5%
	Every two days	30	36.6%
	More than that	18	21.9%
**Annual examination of cats**	Yes	76	92.7%
	No	6	7.3%
**Washing hands before eating**	No	45	8.4%
	Yes, with water only	171	32.1%
	Yes, with water and hand soap	317	59.5%
**Washing fruits and vegetables before eating**	No	9	1.7%
	Yes, with water only	426	79.9%
	Yes, with water and hand soap	98	18.4%
**Eating medium-cooked meat**	Never	386	72.4%
	Rarely	92	17.3%
	Sometimes	48	9.0%
	Often	7	1.3%

**Table 5:** Correlations between having toxoplasmosis and some variables (age, social status, raising cats, washing hands before eating, washing fruits and vegetables before eating, and eating medium-cooked meat) with chi-square test.

Variable	Group	Have You Ever Had Toxoplasmosis?			(Chi-Square Test)	
		Yes (6; 1.1%)	No (527; 98.9%)	Total (533; 100%)	Pearson Chi-Square	*p*-Value
**Age (*n*; %)**	18–25	2; 33.3%	373; 70.8%	375; 70.4%	6.501	0.039
	26–40	3; 50%	74; 14.0%	77; 14.4%		
	41–60	1; 16.7%	80; 15.2%	81; 15.2%		
**Social status** **(*n*; %)**	Married	3; 50%	122; 23.2%	125; 23.5%	2.371	0.124
	Unmarried	3; 50%	404; 76.8%	407; 76.5%		
**Raisaing cats (*n*; %)**	Yes	1; 16.7%	81; 15.4%	82; 15.4%	0.008	0.930
	No	5; 83.3%	446; 84.6%	451; 84.6%		
**Washing hands before eating (*n*; %)**	No	0; 0.0%	45; 8.5%	45; 8.4%	0.568	0.753
	With water only	2; 33.3%	169; 32.1%	171; 32.1%		
	With water and soap	4; 66.7%	313; 59.4%	317; 59.5%		
**Washing fruits and vegetables before eating (*n*; %)**	No	0; 0.0%	9; 1.7%	9; 1.7%	4.081	0.130
	With water only	3; 50%	423; 80.3%	426; 79.9%		
	With water and soap	3; 50%	95; 18.0%	98; 18.4%		
**Eating medium-cooked meat (*n*; %)**	Never	3; 50.0%	383; 72.7%	386; 72.7%	11.710	0.008
	Rarely	1; 16.7%	91; 17.3%	92; 17.3%		
	Sometimes	1; 16.7%	47; 8.9%	48; 9.0%		
	Often	1; 16.7%	6; 1.1%	7; 1.3%		

## Data Availability

The raw data supporting the conclusions of this article will be made available by the authors on request.
